# Effect of Lymphadenectomy on Survival in Early-Stage Type II Endometrial Carcinoma and Carcinosarcoma

**DOI:** 10.1155/2020/1295613

**Published:** 2020-04-10

**Authors:** Dogan Vatansever, Hamdullah Sozen, Gulcin Sahin Ersoy, Burak Giray, Samet Topuz, A. Cem Iyibozkurt, Yavuz Salihoglu

**Affiliations:** ^1^Department of Obstetrics and Gynecology, Gynecologic Oncology Division, Koc University School of Medicine, Istanbul, Turkey; ^2^Faculty of Medicine, Department of Obstetrics and Gynecology, Istanbul University, Istanbul, Turkey; ^3^Department of Obstetrics and Gynecology, Kartal Dr. Lutfi Kirdar Education and Research Hospital, Istanbul, Turkey; ^4^Department of Obstetrics and Gynecology, Zeynep Kamil Education and Research Hospital, Istanbul, Turkey

## Abstract

**Purpose:**

We aimed to investigate whether systematic pelvic and paraaortic lymph node dissection delivers any survival advantage in a subgroup of patients with type II endometrial carcinoma and carcinosarcoma.

**Methods:**

We evaluated 135 patients with clinically early-stage (Stage I-II) type II endometrial carcinoma and carcinosarcoma who underwent systematic pelvic and paraaortic lymph node dissection or who did not undergo any lymph node dissection.

**Results:**

Overall survival (OS) and recurrence-free survivals (RFS) were significantly longer in the systematic lymph node dissection group (hazard ratio 0.28, 95% CI 0.13–0.62 *p*=0.002 for OS and hazard ratio 0.31, 95% CI 0.14–0.69 *p*=0.004 for RFS). Multivariate analysis showed that lymph node dissection, age, lymph node metastasis, and adjuvant therapy were independent prognostic variables of OS and RFS.

**Conclusions:**

Systematic pelvic and paraaortic lymph node dissection independently and significantly prolongs the survival of patients with early-stage type II endometrial carcinoma and carcinosarcoma.

## 1. Introduction

Systematic pelvic and paraaortic lymph node dissection (LND) is one of the cornerstones of surgical treatment of endometrial cancer (EC) [1]. However, the survival benefit of LND is still controversial for early-stage (stage I-II) disease [1–7]. Data from two randomized trials failed to show any therapeutic benefit of LND in early-stage EC [8, 9]. Nevertheless, these trials have been criticized for the low number of harvested lymph nodes, lack of systematic paraaortic LND, the relatively high number of low-risk patients included in the studies, and the heterogeneous nature of adjuvant therapy modalities [1, 3, 10]. In contrast, retrospective findings suggest that systematic LND improves survival in EC [10, 11]. Type II EC comprises nonendometrioid EC subtypes such as uterine papillary serous carcinoma (UPSC) and uterine clear cell carcinoma (UCC), accounting for 10–15% of all ECs [12]. Uterine carcinosarcomas (UCSs) are also widely assessed as type II ECs according to the characteristics of the disease, regarding the pattern of spread, histological appearance at sites of metastasis, and tendency to lymphatic and transperitoneal spread [1, 13]. Systematic pelvic and paraaortic LND widely performed as a part of complete surgical staging in patients presenting with type II endometrial cancer and UCS's since this type of ECs tend to metastasize outside the uterus, including the pelvic and paraaortic lymph nodes more frequently than type I tumors, even in early-stage disease. However, there is no randomized controlled trial, and there is only scarce data in the literature regarding the therapeutic role of LND in this specific group of patients. Most of the patients with type II EC undergo adjuvant treatment; thereby, the benefit of systematic LND on survival of this particular cohort of patients is still a debate. In this retrospective analysis, we compared the survival of two cohorts of patients with a diagnosis of early stage (Stage I-II) type II EC and UCS surgically staged with complete systematic LND (LND+). We also investigated the effect of adjuvant treatment on survival of these two cohorts.

## 2. Materials and Methods

This was an Institutional Review Board-approved retrospective study conducted at Istanbul University School of Medicine, Department of Gynecological Oncology. The study time period was 1998–2013. We searched for patients treated at our institution with a diagnosis of endometrial cancer from the tumor registry. The inclusion criteria consisted of patients with UPSC, UCC, and UCS that had stage I-II disease clinically and radiologically. All of the histopathological investigations were performed by the same two gynecopathologists. The patients were divided into two groups: LND (+) and LND (−). The patients in the LND+ group underwent comprehensive surgical staging consisting of total hysterectomy, bilateral salpingo-oopherectomy, omentectomy, and systematic pelvic and paraaortic LND. The patients in the LND− group also underwent the same surgical staging procedure without any LND. The decision to perform LND and the extent of LND performed was at the discretion of the surgeon. Most of the patients in LND− group have been operated in the first half of study time period. All of the surgeries were performed by the same gynecologic oncology surgical team with standardized surgical methods. The exclusion criteria were (1) endometrioid histology; (2) stage III or IV disease except clinically and radiologically stage I-II disease found to be stage IIIC after surgical staging; (3) presence of synchronous cancers; (4) administration of preoperative chemotherapy or radiotherapy; and (5) severe chronic diseases (chronic heart, renal, or lung disease) and other severe comorbidities. For patients in the LND+ group, the extent of pelvic lymphadenectomy was the psoas muscle laterally, middle of common iliac artery proximally, deep circumflex iliac vein distally, and obturator nerve inferiorly. Bilaterally, all of the lymphatic tissue surrounding the common iliac artery, external iliac artery and vein, internal iliac artery and vein, and lymph nodes in the obturator fossa were dissected. Paraaortic lymphadenectomy was performed with dissection of all lymphatic tissue anterior, posterior, and lateral to the abdominal aorta and inferior vena cava along with the interaortocaval lymph nodes up to the level of the left renal vein. The revised FIGO 2009 staging for endometrial carcinoma was used to classify all patients [14]. After the surgical staging, all of the patients were discussed in the tumor board and it was decided either to give radiotherapy (RT) alone (whole pelvic radiotherapy ± brachytherapy), chemotherapy (CT) alone, or a combination therapy of both (RT and CT) in an adjuvant setting while for some, the decision was only to observe. Patient preferences were also a factor in the decision for adjuvant therapy. We administered four cycles of chemotherapy with a regimen that consisted of carboplatin-paclitaxel to the patients with a diagnosis of UPSC and UCC and ifosfamide-paclitaxel regimen to the patients with a diagnosis of UCS. All of the patients were followed at our institution. Recurrences were detected via physical examination, laboratory studies, and radiological imaging modalities and confirmed by biopsies. Demographic information, clinicopathological variables, and survival data were abstracted from patient medical records. The primary objective of the study was to evaluate the effect of none (LND− group) versus systematic pelvic and paraaortic lymphadenectomy (LND+ group) on overall survival (OS). The secondary objective was to evaluate recurrence-free survival (RFS). OS was defined as the time from surgery to death from any cause and death related to endometrial carcinoma, respectively. RFS was defined as the time from surgery to the first event of recurrent disease.

### 2.1. Statistical Analysis

The Mann–Whitney *U* test was used to determine the statistical significance of the data without normal distribution, and the variables were presented as median and interquartile range (IQR). For categorical data, the chi-square test was used and the variables were presented as percentages. Survival curves were established according to the Kaplan–Meier method and compared using the log-rank test. A Cox regression modeling was used to select the risk factors for prognosis with hazard ratios including variables that showed statistically significant difference with univariate analyses. Statistical analyses were performed using Statistical Package for the Social Sciences (SPSS) version 17 (SPSS Inc., Chicago, IL, USA). In this study, the level of significance was accepted as *p* < 0.05.

## 3. Results

**Figure 1:**
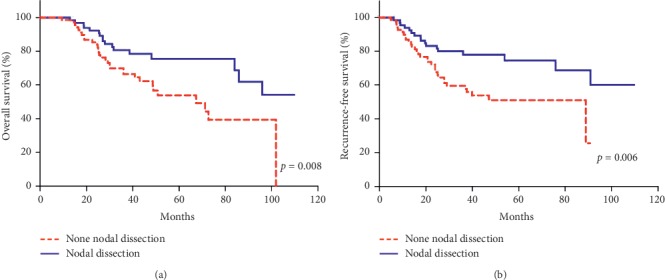
Kaplan–Meier analysis of overall (a) and recurrence-free (b) survival for patients with type II endometrial carcinoma according to lymph node dissection.

One hundred and thirty-five patients meeting the inclusion criteria were enrolled in this study. Clinical and pathological characteristics of patients are summarized in Table 1. Sixty-nine (51%) patients were treated without lymph node dissection (LND−), whereas 66 (49%) patients underwent systematic pelvic and paraaortic lymph node dissection (LND+). The median age was 64 (IQR 54.50–73.50) in the LND− group and 62.5 (IQR 54.75–69) in the LND+ group (*p*=0.347). There was no statistically significant difference between the two groups regarding the distribution of prognostic variables except the surgical stage (*p* < 0.0001). All of the patients had clinically early stage disease without any radiologically suspicious metastatic lymph nodes or extrauterine disease as previously noted. However, in the LND+ group, 16 patients were discovered who had metastatic lymph node and 6 (37.5%) were staged as 3C1 and 10 (62.5%) were staged as 3C2. The localization of these metastatic lymph nodes was only in the pelvic region in 6 (37.5%), the pelvic and paraaortic region in 8 (50%), and only the paraaortic region in 2 (12.5%) of the patients. One hundred and twenty-one (85%) patients received adjuvant therapy and the distribution of patients according to stage and type of adjuvant treatment is presented in Table 2. There was no significant difference between the two groups in terms of adjuvant therapy as none versus any (RT, CT, or RT + CT) (*p*=0.332) (Table 1). Similarly, we found no statistically significant difference between the two groups when they were compared according to the four subgroups (observation, RT, CT, or RT + CT) of adjuvant treatment modalities (*p*=0.410) (Table 3). Moreover, we performed the Kaplan–Meier analysis of survival in the LND+ and LND− groups, and we found that OS and RFS were significantly longer in the LND+ group of patients (*p*=0.008 for OS and *p*=0.006 for RFS) (Figures 1(a) and 1(b)). In addition, LND had an advantage of 9.7% for 5-year OS and 8.9% for 5-year RFS in the LND+ group compared to the LND− group (Table 4). Cox regression analysis revealed that LND, younger age, earlier stage, and adjuvant therapy were associated with an independent and significant improvement in OS and RFS (Table 5).

## 4. Discussion

The analysis of our data has suggested that systematic lymph node dissection including bilateral pelvic regions and paraaortic region may improve OS and RFS in patients with early-stage (Stage I-II) type II endometrial carcinoma and UCS. We also found that lymph node dissection and adjuvant therapy were independently and positively correlated with prolonged survival of patients, while increased age and stage were negatively correlated. The role of systematic lymphadenectomy and its therapeutic effect on endometrial carcinoma have been investigated in several studies [8–12]. However, the therapeutic significance of LND is still a great debate. There are two randomized clinical trials that failed to show a therapeutic benefit of LND [8, 9]. The ASTEC trial has been criticized for the number of harvested lymph nodes (less than 10 in 35% of patients) and lack of a standardized systematic pelvic and paraaortic LND [8]. In the Italian trial, Benedetti et al. [9] also failed to show any therapeutic advantage of LND. They reported higher harvested lymph node numbers. However, the Italian study has also been criticized for the lack of a systematic paraaortic LND. The number of low-risk patients included in both of these studies was found to be relatively high, and the adjuvant therapy modalities were heterogeneous. In contrast, retrospective data supports the therapeutic effect of LND in patients with endometrial carcinoma [10, 11]. Smith et al. [11] evaluated the data of 42,184 patients with EC through a Surveillance, Epidemiology, and End Results analysis and showed that LND was associated with improved OS and DSS with hazard ratios (HR) of 0.81 and 0.78, respectively. In the SEPAL study, which was a well-designed retrospective study, 671 patients with EC were separated into two groups according to the presence of systematic paraaortic LND, and it was shown that paraaortic LND had survival benefits for patients in the intermediate or high-risk groups. Nevertheless, there were only 21 patients in the pelvic LND group and 25 patients in the pelvic and paraaortic LND group diagnosed as UPSC and UCC, which accounted for 6.9% of the study population [10]. Vogel et al. [12] investigated the role of paraaortic LND in patients with UPSC and UCC in a retrospective study as a secondary outcome, and they could not show any significant therapeutic effect. However, they concluded that their results may have been biased because of the heterogeneous distribution of adjuvant therapy modalities [12]. In our study, we investigated the data of 135 patients with a diagnosis of UPSC, UCC, or UCS. The overwhelming majority of patients were diagnosed as UPSC or UCC (115 patients, 85.2%). All of the patients were in the early clinical stage of the disease and were evaluated by radiologic imaging techniques to exclude those with extrauterine spread of the disease and pathological lymph nodes. The surgical treatment technique was identical for both groups of patients except the systematic pelvic and paraaortic LND performed for patients in the LND+ group. There was no significant difference in the distribution of clinicopathological parameters, including age, histologic type, myometrial invasion, cervical involvement, and lymphovascular space invasion (LVSI) between the two groups. The only significant differences between the LND− and LND+ groups were the distribution of FIGO stages of patients. This can be attributed to the study design, since we could not detect patients with lymph node metastasis in the LND− group. Even though there was no significant difference (*p*=0.410), 47% of the LND+ group received RT + CT vs. 33% of the patients in LND− group. The patients with a severe chronic disease or comorbidity that might affect the surgeons' decision on lymph node dissection were excluded from the study in order to minimize selection bias. As a result, the patients in the two groups were found to be clinically comparable and there was no significant difference between the LND− and LND+ groups of patients except the presence of systematic LND in the surgical treatment algorithm. The number of harvested lymph nodes was reported to be important for the therapeutic effect of LND [1]. In a meta-analysis of nine trials, Kim et al. [15] showed that removal of ≥10-11 lymph nodes was related to improved OS in patients with intermediate and high-risk endometrial cancer. The median number of harvested lymph nodes in the LND+ group was 19 (15–41) in our study. The extent of paraaortic LND is also important. Mariani et al. [16] evaluated endometrial carcinoma patients excluding the low-risk group and reported that 33% of the metastatic lymph nodes were in the pelvic region only, whereas 16% had only paraaortic metastasis and 51% had pelvic and paraaortic metastasis, which indicates that 67% of patients had a metastatic lymph node in the paraaortic region. Additionally, 77% of metastatic lymph nodes in the paraaortic region were located between the inferior mesenteric artery and the renal vein [16]. Turan et al. [17] also reported similar rates of metastatic lymph node locations as 38% only pelvic, 45% pelvic and paraaortic, and 16% only paraaortic in their study. Odagiri et al. [18] evaluated the recurrence pattern and survival of 147 patients with stage IIIC1 to IIIC2 endometrial carcinoma, and they concluded that recurrence is the only independent prognostic factor for survival and the number of harvested lymph nodes along with paraaortic lymph node metastasis predicted the recurrence risk. Therefore, they suggested a systematic pelvic and paraaortic LND in patients with a high risk of lymph node metastasis [18]. The number of metastatic lymph nodes was 16 (24%) in the LND+ group and the anatomic locations were only pelvic in 6 (37.5%) patients, pelvic and paraaortic in 8 (50%) patients, and only paraaortic in 2 (12.5%) patients in our study, which is in line with these studies. The adjuvant therapy and the treatment modalities chosen are important factors for the outcome of patients, in addition to surgical technique. Viswanathan et al. [19] suggested an OS benefit for adjuvant chemotherapy with a paclitaxel-platinum regimen and a recurrence-free survival benefit for adjuvant radiotherapy in serous cancers. In addition, in the PORTEC-3 trial, a significant improvement in both 5-year overall survival and failure-free survival was found for women with serous cancers treated with chemoradiotherapy [20]. However, Hogberg et al. [21] failed to show any survival benefit of combination therapy with CT and RT in their subgroup analysis of NSGO/EORTC and MANGO-ILIADE trials for patients with UPSC and UCC. In the SEPAL study, 77% of the patients in the pelvic and paraaortic LND group received CT whereas 45% of patients who underwent only pelvic LND received CT [10]. We found in our study that adjuvant therapy was an independent prognostic factor for OS and RFS in early stage type II endometrial cancers. There was no significant difference between the groups in our study, whether comparing no adjuvant treatment with any type of adjuvant treatment or comparing subgroups of treatments (Tables 1 and 3). The limitations of our study were that our study was a retrospective cohort analysis and prone to selection bias even though the accompanying prognostic factors were distributed homogenously between LND+ and LND− groups of patients. Additionally, even though the two groups of patients did not show any significant difference in distribution of adjuvant therapy modalities, 47% of the LND+ group received RT + CT vs. 33% of the patients in LND− group. To the best of our knowledge, this is the first study comparing systematic pelvic and paraaortic LND with no LND in type II endometrial carcinomas, and we believe that the data we analyzed is still worthwhile in the absence of randomized controlled trials.

## 5. Conclusions

We demonstrated that systematic LND may increase OS and RFS in this group of patients. Additionally, we showed that adjuvant therapy is also a positive independent prognostic factor for patients with type II endometrial carcinoma, whereas age over 65 years and stage were negatively correlated with survival. Systematic pelvic and paraaortic LND is a cardinal step of surgical treatment of patients with type II endometrial carcinomas, and we have been able to demonstrate that systematic pelvic and paraaortic LND significantly improves survival in patients with type II endometrial carcinoma and UCS in our cohort of patients.

## Figures and Tables

**Table 1:** Clinical and pathological characteristics of patients with type II endometrial carcinoma.

	No nodal dissection (*n* = 69)	Systematic nodal dissection (*n* = 66)	*p* value
Age (year) (median) (IQR) surgical stage	64 (54.50–73.50)	62.5 (54.75–69.00)	0.347
IA	34 (49%)	30 (46%)	**<0.0001**
IB	24 (35%)	14 (21%)	
II	11 (16%)	6 (9%)	
IIIC	0 (0%)	16 (24%)	

Histology			0.835
Carcinosarcoma	10 (14%)	10 (15%)	
Serous adenocarcinoma	42 (61%)	37 (56%)	
Clear cell adenocarcinoma	17 (25%)	19 (29%)	

Adjuvant therapy			0.332
None	10 (14%)	6 (9%)	
Any	59 (86%)	60 (91%)	

Myometrial invasion			0.318
<50%	37 (54%)	41 (62%)	
≥50%	32 (46%)	25 (38%)	

Cervical involvement			0.325
Negative	54 (78%)	56 (85%)	
Positive	15 (22%)	10 (15%)	

Lymphovascular space invasion			0.444
Negative	31 (45%)	34 (51%)	
Positive	38 (55%)	32 (49%)	

Lymph node metastasis^*∗*^			
Negative	—	50 (76%)	
Positive	—	16 (24%)	

Follow-up period (m, median) (range)	39.9 (9–101)	39.6 (12–109)	0.162

^*∗*^Data not calculated. IQR: interquartile range, m:months. Median age of the group = 63 years.

**Table 2:** Adjuvant treatment by stage in patients with type II endometrial carcinoma.

Stage	Observation	Only RT	Only CT	RT + CT
IA	10 (16%)	20 (31%)	16 (25%)	18 (28%)
IB	6 (16%)	14 (37%)	2 (5%)	16 (42%)
II	0 (0%)	8 (47%)	4 (24%)	5 (29%)
IIIC	0 (0%)	1 (6%)	0 (0%)	15 (94%)

Data are number of patients (%). RT: radiotherapy, CT: chemotherapy.

**Table 3:** Distribution of adjuvant therapy across patients with type II endometrial carcinoma according to lymph node dissection.

Lymph node dissection	Observation	Only RT	Only CT	RT + CT	*p*
None	10 (15%)	24 (35%)	12 (17%)	23 (33%)	0.410
Yes	6 (9%)	19 (29%)	10 (15%)	31 (47%)	

**Table 4:** Overall and recurrence-free survival of patients with type II endometrial carcinoma.

	No nodal dissection (*n* = 69)	Systematic nodal dissection (*n* = 66)
Overall survival		
Died	31 (45%)	17 (26%)
3 years	66.5%	78.5%
5 years	44.3%	54.0%

Recurrence-free survival		
Relapsed or died	32 (46%)	17 (26%)
3 years	59.5%	78.0%
5 years	51.1%	60.0%

Data are number of patients (%) or percentage survival.

**Table 5:** Multivariate analysis of overall and recurrence-free survival by risk factor in type II endometrial cancer.

	Overall survival	*p*	Recurrence-free survival	*p*
	Hazard ratio (95% CI)		Hazard ratio (95% CI)	
LND				
None	1.00		1.00	
Yes	0.28 (0.13–0.62)	**0.002**	0.31 (0.14–0.69)	**0.004**

Age-group (years)				
≤65	1.00		1.00	
>65	3.42 (1.71–6.83)	**<0.0001**	2.80 (1.47–5.33)	**0.002**

Stage				
IA	1.00		1.00	
IB	0.74 (0.33–1.66)	0.473	1.11 (0.49–2.50)	0.793
II	1.83 (0.73–4.56)	0.195	2.98 (1.20–7.36)	**0.018**
IIIC	4.76 (1.58–14.38)	**0.006**	5.23 (1.66–16.49)	**0.005**

Adjuvant therapy				
None	1.00		1.00	
Any	0.22 (0.09–0.51)	**<0.0001**	0.21 (0.09–0.46)	**<0.0001**

## Data Availability

The data used to support the findings of this study are available from the corresponding author upon request.
